# Diagnostic Implications of an Elevated Troponin in the Emergency Department

**DOI:** 10.1155/2015/157812

**Published:** 2015-04-16

**Authors:** Maame Yaa Yiadom, Petr Jarolim, Cathy Jenkins, Stacy E. F. Melanson, Michael Conrad, Joshua M. Kosowsky

**Affiliations:** ^1^Department of Emergency Medicine, Vanderbilt University, Nashville, TN 37232, USA; ^2^Department of Pathology, Brigham and Women's Hospital, Harvard Medical School, Boston, MA 02115, USA; ^3^Department of Biostatistics, Vanderbilt University, Nashville, TN 37203, USA; ^4^Department of Emergency Medicine, Brigham and Women's Hospital, Boston, MA 02115, USA

## Abstract

*Objective*. To determine the proportion of initial troponin (cTn) elevations associated with Type I MI versus other cardiovascular and noncardiovascular diagnoses in an emergency department (ED) and whether or not a relationship exists between the cTn level and the likelihood of Type I MI. *Background*. In the ED, cTn is used as a screening test for myocardial injury. However, the differential diagnosis for an initial positive cTn result is not clear. *Methods*. Hospital medical records were retrospectively reviewed for visits associated with an initial positive troponin I-ultra (cTnI), ≥0.05 *μ*g/L. Elevated cTnI levels were stratified into low (0.05–0.09), medium (0.1–0.99), or high (≥1.0). Discharge diagnoses were classified into 3 diagnostic groups (Type I MI, other cardiovascular, or noncardiovascular). *Results*. Of 23,731 ED visits, 4,928 (21%) had cTnI testing. Of those tested, 16.3% had initial cTnI ≥0.05. Among those with elevated cTn, 11% were classified as Type I MI, 34% had other cardiovascular diagnoses, and 55% had a noncardiovascular diagnosis. Type I MI was more common with high cTnI levels (41% incidence) than among subjects with medium (9%) or low (6%). *Conclusion*. A positive cTn is most likely a noncardiovascular diagnosis, but Type I MI is far more common with cTnI levels ≥1.0.

## 1. Introduction

Myocardial infarction (MI) in emergency department (ED) patients is a frequently suspected but infrequently made diagnosis. In the ED patient population a troponin serum assay (cTn) is used as a generic screen for myocardial injury [1, 2]. Serum cTn testing is the biomarker of choice to test for myocardial infarction, one of the more critical conditions causing myocardial injury. An elevated troponin has been associated with increased morbidity and mortality. So this generally prompts a continued evaluation in hospital [3]. The biochemical specificity of contemporary cardiac troponin assays, a troponin I (cTnI) upon initial serum testing in the ED, is greater than 90% for myocardial injury [4, 5]. This permits the early identification of patients with likely myocardial injury. A 2008 study by Keller et al. suggests that the diagnosis of acute myocardial infarction can be made early for many patients with an initial early elevated cTn level in the right clinical context. However, the likelihood that an initial elevated cTnI specifically represents Type I MI is less clear. Troponin based diagnostic decision-making primarily pertains to patients with potential non-ST-elevation MI (NSTEMI), since the diagnosis of STEMI is made by EKG. However, troponin testing still has a role in differentiating cases of MI from those where ST elevations on EKG may be attributed to other causes.

Even among causes of MI, there are several other mechanisms of injury to be noted. In these cases, acute management focuses on treating the underlying causes, rather than medication or procedural coronary intervention. This diversity was recognized within the universal definition of myocardial infarction as classified by the international consortium of cardiology associations. (see Table 1) [6]. Type I MI was independently distinguished from other mechanisms of acute myocardial infarction (AMI), as myocardial cell loss from ischemia caused by acute atherosclerotic plaque rupture with thrombus formation within a coronary artery lumen, fissuring, or dissection (i.e., acute coronary syndrome).

The loss of myocardium occurs on the order of minutes; thus a diagnosis of Type I MI prompts an acute shift in focus toward early antiplatelet and anticoagulant therapy with the possibility of urgent percutaneous coronary intervention (PCI). Little epidemiological data exists to guide practitioners in gauging the suspicion for Type I MI, amongst other causes, when the cTn is elevated in the context of a broad differential diagnosis. This situation is typical in the ED. The first objective of this study is to assess the frequency and implications of the diagnosis of Type I MI in an undifferentiated ED patient population. In addition, it is commonly assumed that the higher the cTn elevation, the greater the likelihood of Type I MI. A 2001 substudy by Lindahl et al. [7] suggested that this was the case with a cTnT assay. We challenge this hypothesis using a cTnI assay.

## 2. Materials and Methods

### 2.1. Study Population

This study was performed at a tertiary care academic medical and trauma center with 56,000 adult ED patient visits a year using a sensitive contemporary troponin biomarker for the evaluation of MI. Prior to the initiation of this retrospective chart review, it was approved by the hospital human research committee. The study period was March 1–July 31, 2007. The study population included all patients ≥18 years of age with a positive (≥0.05 *μ*g/L) initial ultrasensitive cTnI (TnI-Ultra, Siemens Healthcare Diagnostics, Tarrytown, New York) [8, 9] during their ED evaluation. Patients presenting in cardiac arrest were excluded. Typical practice in the ED at this time was to test appropriate patients for a cTn elevation upon initial evaluation and then 6 hours after presentation. Patients with a cTn elevation at first measurement were included. Results were obtained from the laboratory information system and merged, with the associated patient data from the electronic medical record system, in Microsoft Excel.

### 2.2. Adjudication of the Final Diagnosis

The final hospital discharge diagnosis was confirmed through a review of each patient's ED and in-hospital record by a senior emergency medicine attending physician. Patients were categorized into diagnostic groups as either Type I MI (on the basis of care consistent with MI management including urgent revascularization, sustained antithrombotic therapy, or percutaneous coronary intervention during the hospital course), other cardiovascular diagnoses (including dysrhythmias, pulmonary embolism, and congestive heart failure), or a noncardiovascular diagnosis. In addition, deaths and the incidence of urgent revascularization were noted.

### 2.3. Analysis of Troponin Results

TnI results were stratified as low (0.05–0.09 *μ*g/L), medium (0.1–0.99 *μ*g/L), and high (≥1.0 *μ*g/L). Frequencies of primary diagnoses were determined by troponin strata, major diagnostic grouping, and the total population outcomes. The association of diagnostic grouping and categorized cTn level was assessed using Pearson's chi-square test. All statistical analyses were performed using the programming language R, Version 3.1.2 (http://cran.r-project.org/).

## 3. Results

**Figure 1:**
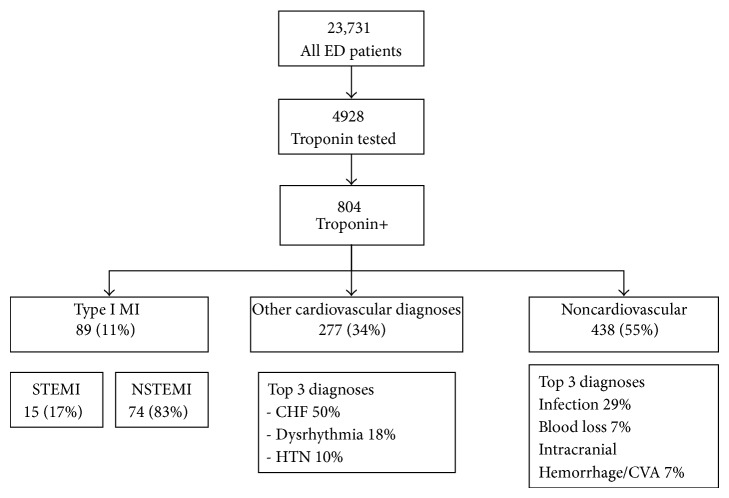
Study population and diagnostic groups.

During the 5-month study period, 23,731 patients were seen in the ED of which 4,928 (21%) had a cTnI testing as part of their ED evaluation (see Figure 1). Among the tested subjects, 804 had an elevated initial cTnI. This represents 3.4% of all patients seen and 16.3% of those that had a cTnI ordered. The leading causes of an elevated cTnI across all diagnostic groups were from congestive heart failure (17%), infection (16%), dysrhythmia (6%), and blood loss (4%). In the low troponin level strata (0.05–0.9 ng/mL) were 383 patients (48%), 339 (42%) fell into the medium strata (0.1–0.99 ng/mL), and 82 (10%) fell into the high strata (≥1.0) (See Table 2). The type of primary diagnosis was significantly associated with categorized cTnI (*P* < 0.001). Eleven percent had a final diagnosis of Type I MI. This was only 1.8% of those who had a troponin level checked and 0.03% of all patients evaluated. Fifteen (17%) had ST-elevation MI and 74 (83%) had non-ST-elevation MI. Eight percent had urgent revascularization.

Among those with Type I MI, 26% had low troponin levels, 36% had medium, and 38% had high. Sixty-six patients (74%) underwent urgent revascularization, and 6% died. These deaths accounted for a minority (7%) of all deaths. Other cardiovascular diagnoses occurred in 277 (35%) of patients with an elevated cTnI. The most frequent diagnoses in this group were congestive heart failure (53%), dysrhythmia (18%), and hypertension (9%). Ten (4%) of these patients died accounting for 12% of all deaths. Noncardiovascular diagnoses were identified for 438 (55%) patients. The top diagnoses in this group were infection (29%), blood loss (7%), and intracranial hemorrhage or stroke (7%). Seventy (16%) of these patients died and accounted for 82% of all deaths.

**Figure 2:**
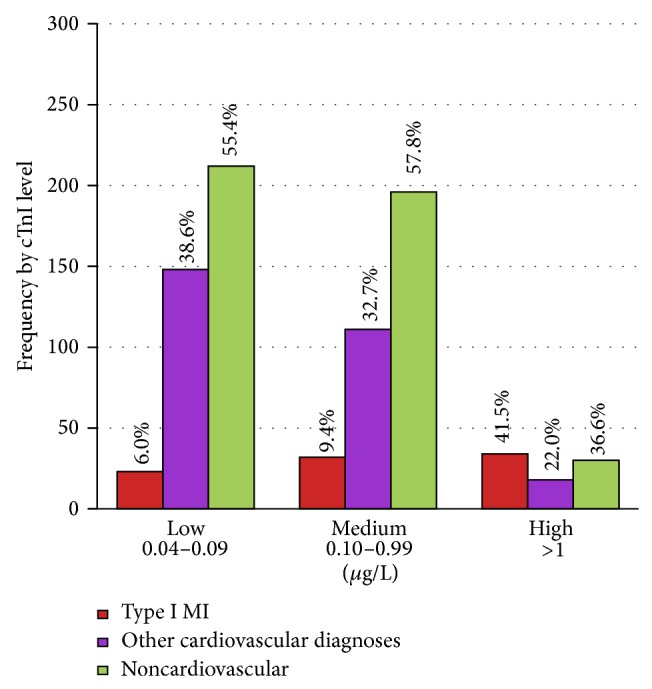


In examining the troponin level (see Table 3) we found that within the low strata 6% had a final diagnosis of Type I MI, 5% underwent urgent revascularization, 39% had other cardiovascular diagnoses, 55% of patients had a noncardiovascular diagnosis (see Figure 2), and 7% died. These deaths were 33% of all deaths. Within the medium strata, 9% had an end diagnosis of Type I MI, 6% of the group experienced urgent revascularization, 33% had other cardiovascular diagnoses, 58% had a noncardiovascular diagnosis, and 12% died. These deaths accounted for 47% of all deaths. The leading diagnoses within this group were dysrhythmia with atrial fibrillation accounting for all cases. In the high strata the leading diagnoses were Type I MI, AICD firing, and blood loss. Within this group 41% of patients had Type I MI, 34% had urgent revascularization, 22% had other cardiovascular diagnoses, 37% had a noncardiovascular diagnosis, and 21% of all patients with a high troponin died. This was 20% of all deaths.

## 4. Discussion

This study demonstrates that myocardial infarction is the cause of troponin elevation in a minority of cases. The striking finding from the study is the diversity (see Figure 1) within the differential diagnosis for an elevated cTnI in an undifferentiated patient population. The majority of deaths among those with a positive troponin (82%) were attributed to noncardiovascular diagnoses. Our results support prior work that has identified the incidence of MI in this population to be between 9 and 13% and the troponin elevations to be mostly attributed to noncardiac diagnoses [10, 11]. However, we also found that patients with high initial troponin levels had a much higher incidence of Type I MI (see Figure 2). The result of the first troponin test in the ED helps gauge the likelihood of admission versus discharge and the likely focus of early in-hospital care. Unlike other MI types, treatment is focused on early antithrombotic coronary artery therapies. Thus the likelihood of a positive initial troponin being Type I MI is of high importance in priming these time-sensitive interventions.

The specificity of a cTn elevation being attributed to Type I MI increases with serial testing. Because of this, the 2014 American College of Cardiology/American Heart Association guidelines for the diagnosis and management of NSTEMI advise measuring troponin levels at 3–6 hours after symptoms onset and beyond 6 hours for patients with a moderate to high risk [12]. However, an ideal ED evaluation is completed at 4–6 hours, and the report of symptom onset is not always reliable upon early patient evaluation in the ED. This prompts many physicians to use ED arrival as their start time for serial testing. As a result, decisions on whether to admit or discharge patients, particularly when the initial troponin is positive, are often made before serial testing is completed.

In addition, many ED patients are undifferentiated, thus undergoing simultaneous evaluations for other cardiovascular and noncardiovascular conditions that extend into their in-hospital stay. A troponin elevation is only a marker of myocardial injury, which can result from multiple mechanisms that cause myocyte death [13] including ischemia from acute coronary syndrome (which includes STEMI and NSTEMI), surgical trauma [14], mechanical trauma [15], poorly understood neurohormonal and inflammatory processes, and systemic demand [16]. Insight into the likely differential diagnosis of an elevated troponin can help receiving in-hospital teams identify and target the true cause of myocardia injury with more clarity.

The findings of this study shed light on the likely differential diagnosis when myocardial injury is demonstrated by an increased troponin level. Specifically, we found that just under half of all positive tests were attributed to CHF, infection, dysrhythmia, or blood loss. The quest for increased sensitivity in identifying potential MI in patients has led to biomarkers that have dramatically increased the number of patients with a positive test. This study was performed at time when this institution was switching from a cTnI assay to the more sensitive cTnT. Both the department of pathology and emergency medicine wanted to better understand the end diagnostic nature of false positive tests when a troponin was used to screen for myocardial infarction as a subset of myocardial injury and to better understand the alternative diagnoses to consider with a positive test.

Regardless of the etiology, an elevated cTnI result often prompts a hospital admission for further investigation [17]. Serial troponin testing is more sensitive for the ultimate diagnosis of Type I MI [18]. However, two 2009 studies by Gudmundsson et al. [3] and Amsterdam et al. [12] note the diagnostic sensitivity of an initial ultrasensitive or high-sensitive ED cTnI assay in the early diagnosis of Type I MI. Biochemical evidence of myocardial injury in the context of concerns for MI will prompt an in-hospital stay, because this finding increases the risk of a negative outcome regardless of the etiology [3]. In our study we observed that mortality trended with the level of troponin elevation.

## 5. Study Limitations

Some limitations of our study should be considered. This is a retrospective study including data collected from a heterogeneous mix of patients. Each patient had a cTnI blood level sent by a heterogeneous group of emergency medicine physicians that were not using a standard protocol to screen for myocardial injury or infarction. There is no strict policy or protocol for when to test for a troponin I level in this institution. However, all troponins require a physician order to be processed. We did not follow the final diagnosis of patients whose cTnIs were negative during their ED stay or any associated hospital stay. This prevented us from identifying the negative and positive predictive values of the initial ED troponin in the population of patients being evaluated for Type I MI and we did not assess the implications of serial troponin testing as this was outside the scope of this study. Many “other cardiovascular” diagnoses qualify as MI. However, they are typically Type II MI which is from increased oxygen demand or decreased oxygen supply to the demand or restrictions of another cardiovascular pathology. However, given our intent to identify those patient who will receive early acute coronary syndrome intervention this study is limited to the identification of individuals with Type I MI.

## 6. Conclusion

Concerns for potential MI may be the primary reason for testing, but a minority of patents with a positive result had Type I MI as their final diagnosis. Most common diagnoses were congestive heart failure, infections, dysrhythmias, and blood loss. In addition, the majority of deaths were due to alternative diagnoses with most falling in the noncardiovascular diagnostic group. This supports existing evidence that myocardial injury is a marker of increased morbidity and mortality. We also observed an increasing trend in mortality correlating to the level of troponin elevation. In comparing troponin strata, Type I MI was more common in the medium strata than the low strata and significantly more present in the high troponin strata. Overall, a patient who has a positive cTnI will most likely have other cardiovascular or noncardiovascular diagnoses since only 11% of all patients had Type I MI.

## Figures and Tables

**Table 1:** Types of myocardial infarction (MI).

Type	Definition
I	Infarction due to ischemia from a primary coronary event such as atherosclerotic plaque rupture and thrombus formation, arterial wall erosion, fissuring, or dissection

II	Infarction secondary to ischemia from either increased oxygen demand or decreased supply (coronary artery spasm, hypotension, hypertension, anemia, and dysrhythmia)

III	Sudden cardiac death/arrest with symptoms suggestive of STEMI or thrombus in a coronary artery found on angiography or autopsy

IVa	Infarction resulting from percutaneous coronary intervention (PCI)

IVb	Infarction from stent thrombosis

V	Infarction due to ischemia related to coronary artery bypass grafting

**Table 2:** Positive troponin population data analysis by troponin level strata.

	*N*	Low	Medium	High	Combined	*P* value
		*N* = 383	*N* = 339	*N* = 82	*N* = 804	
Age	804	59 72 82	58 70 81	57 67 80	58 71 81	0.22^1^
Sex	804					0.95^2^
Female		46% (177)	47% (160)	48% (39)	47% (376)	
Male		54% (206)	53% (179)	52% (43)	53% (428)	
First cTnI	804	0.05 0.06 0.08	0.12 0.20 0.35	1.73 3.00 6.29	0.06 0.10 0.27	<0.001^1^
Type of diagnosis	804					<0.001^2^
Type 1 MI		6% (23)	9% (32)	41% (34)	11% (89)	
Other cardiovascular diagnoses		39% (148)	33% (111)	22% (18)	34% (277)	
Noncardiovascular		55% (212)	58% (196)	37% (30)	54% (438)	
eGFR	758	30 53 75	26 49 72	38 64 85	29 52 75	0.015^1^
CKMB	759	1.2 2.4 4.0	1.6 3.0 5.3	5.1 9.2 21.3	1.6 2.9 5.5	<0.001^1^
Creatinine	758	0.90 1.21 2.00	0.94 1.30 2.30	0.87 1.08 1.60	0.90 1.24 2.04	0.026^1^
MI	804					<0.001^2^
No		94% (360)	91% (307)	59% (48)	89% (715)	
Yes		6% (23)	9% (32)	41% (34)	11% (89)	
Urgent revascularization	804					<0.001^2^
No		95% (364)	94% (320)	66% (54)	92% (738)	
Yes		5% (19)	6% (19)	34% (28)	8% (66)	
Status	804					0.001^2^
Alive		93% (355)	88% (299)	79% (65)	89% (719)	
Died		7% (28)	12% (40)	21% (17)	11% (85)	

*a* *b* *c* represent the lower quartile *a*, the median *b*, and the upper quartile for continuous variables.

*N* is the number of nonmissing values.

Numbers after percents are frequencies.

Tests used: ^1^Kruskal-Wallis test; ^2^Pearson's test.

**Table 3:** Positive troponin population data analysis by diagnostic groups.

	*N*	Type I MI	Other cardiovascular diagnoses	Noncardiovascular	Combined	*P* value
		*N* = 89	*N* = 277	*N* = 438	*N* = 804	
Age	804	57 64 77	57 69 80	60 74 83	58 71 81	<0.001^1^
Sex	804					0.38^2^
Female		40% (36)	46% (128)	48% (212)	47% (376)	
Male		60% (53)	54% (149)	52% (226)	53% (428)	
First cTnI	804	0.09 0.34 2.49	0.06 0.09 0.21	0.06 0.10 0.24	0.06 0.10 0.27	<0.001^1^
First cTnI (categorized)	804					<0.001^2^
Low		26% (23)	53% (148)	48% (212)	48% (383)	
Medium		36% (32)	40% (111)	45% (196)	42% (339)	
High		38% (34)	6% (18)	7% (30)	10% (82)	
eGFR	758	52 73 90	30 51 72	25 50 72	29 52 7	<0.001^1^
CKMB	759	1.9 3.6 9.5	1.6 2.7 4.2	1.5 2.9 5.6	1.6 2.9 5.5	0.003^1^
Creatinine	758	0.80 1.00 1.26	0.90 1.30 2.00	0.98 1.30 2.35	0.90 1.24 2.04	<0.001^1^
MI	804					<0.001^2^
No		0% (0)	100% (277)	100% (438)	89% (715)	
Yes		100% (89)	0% (0)	0% (0)	11% (89)	
Urgent revascularization	804					<0.001^2^
No		26% (23)	100% (277)	100% (438)	92% (738)	
Yes		74% (66)	0% (0)	0% (0)	8% (66)	
Status	804					<0.001^2^
Alive		94% (84)	96% (267)	84% (368)	89% (719)	
Died		6% (5)	4% (10)	16% (70)	11% (85)	

*a* *b* *c* represent the lower quartile *a*, the median *b*, and the upper quartile for continuous variables.

*N* is the number of nonmissing values.

Numbers after percents are frequencies.

Tests used: ^1^Kruskal-Wallis test; ^2^Pearson's test.
